# Age-related decline in peak oxygen uptake: Cross-sectional vs. longitudinal findings. A review

**DOI:** 10.1016/j.ijcrp.2023.200171

**Published:** 2023-01-13

**Authors:** Jon Magne Letnes, Bjarne M. Nes, Ulrik Wisløff

**Affiliations:** a Department of Circulation and Medical Imaging, Norwegian University of Science and Technology, Trondheim, Norway; b Clinic of Cardiology, St. Olavs University Hospital, Trondheim, Norway; c School of Human Movement and Nutrition Science, University of Queensland, Queensland, Australia

**Keywords:** Peak oxygen uptake, Cardiorespiratory fitness, Cardiopulmonary reference data, Exercise testing

## Abstract

Cardiorespiratory fitness is established as an important prognostic factor for cardiovascular and general health. In clinical settings cardiorespiratory fitness is often measured by cardiopulmonary exercise testing determining the gold-standard peak oxygen uptake (VO_2peak_). Due to the considerable impact of age and sex on VO_2peak_, results from cardiopulmonary exercise testing are typically assessed in the context of age- and sex-specific reference values, and multiple studies have been conducted establishing reference materials by age and sex using cross-sectional designs. However, crossectional and longitudinal studies have shown somewhat conflicting results regarding age-related declines of VO_2peak_, with larger declines reported in longitudinal studies. In this brief review, we compare findings from crossectional and longitudinal studies on age-related trajectories in VO_2peak_ to highlight differences in these estimates which should be acknowledged when clinicians interpret VO_2peak_ measurements repeated over time.

Cardiorespiratory fitness (CRF) is established as a powerful marker of present and future health, with higher levels associated with a reduced risk of not only cardiovascular disease and mortality, but also a plethora of other diseases [[1], [2], [3]]. Studies employing machine-learning have shown that age and CRF are the two features with greatest impact on mortality prediction in cardiac rehabilitation settings, outperforming commonly used variables from clinical practice [4]. Still, the potential clinical value of CRF in patient follow-up and preventive settings has received little attention, leading to an initiative from the American Heart Association advocating to increase the uptake of CRF assessment in clinical practice [3]. CRF can be estimated by several methods, including non-exercise methods, but the gold-standard method is direct measurement of peak oxygen uptake (VO_2peak_) by ventilatory gas-analysis during dynamic exercise to voluntary exhaustion [5].

Already in 1938, in a comprehensive work of experimental studies of physical fitness in relation to age, Robinson described that “*the mechanism for supplying and utilizing O*_*2*_ *in exhaustive work are only about 50 per cent as effective in a man of 75 as in a boy of 17*” [6], hence underscoring the importance of considering age when assessing fitness levels. Together with age, both sex and exercise training status are key determinants of CRF, but still age alone explains 30–40% of variation [7,8]. Using age-adjusted reference data is therefore necessary, and a wide variety of studies have published reference data on VO_2peak_ by age and sex over the last couple of decades [9,10]. Comparing with a reference standard is necessary to accurately interpret individual patients’ fitness levels in clinical settings. However, considerable variation in the age-related decline of VO_2peak_ has been reported when comparing studies of crossectional and longitudinal designs, as previously discussed by Hawkins and Wiswell [11]. In the years since that publication a plethora of studies have been published, including large studies assessing longitudinal declines by different age-groups.

Still, the age-related decline in fitness is generally referred to be ∼10% per decade, even though this is a simplification, and may be grossly inaccurate as will be discussed. Therefore, in this brief review we overview the current literature and compare findings from cross-sectional and longitudinal studies on age-related trajectories in VO_2peak_. We highlight differences, and show how these differences may be of importance when performing long-time patient follow-up and when interpreting repeated VO_2peak_/CRF measurements over time. Relevant studies were identified using structured searches in PubMed and by further review of references in the identified publications of interest.

## 1 Age-related declines in cross-sectional studies

**Fig. 1:**
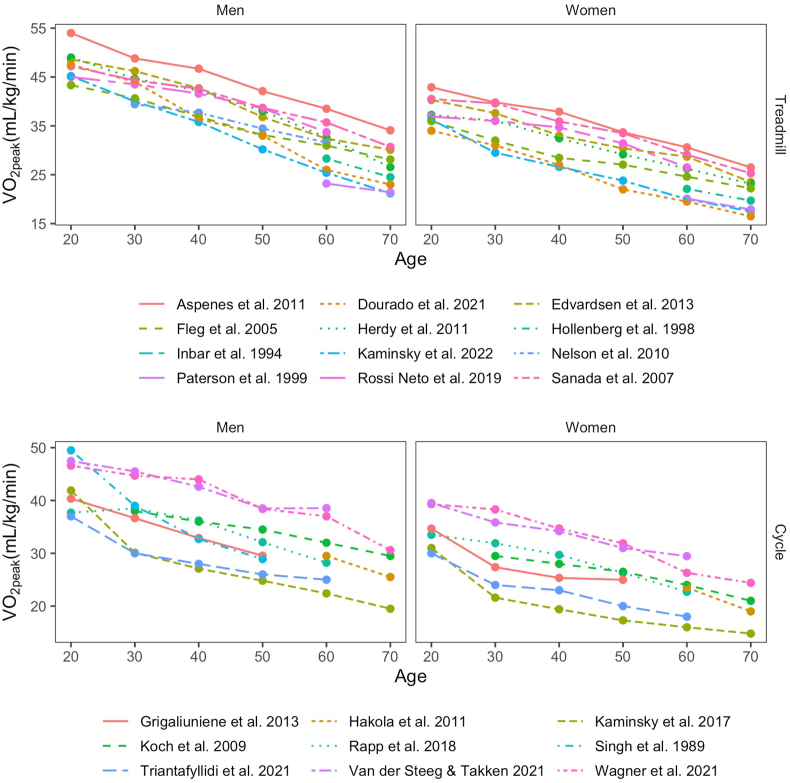
Cross-sectional reference data for VO_2peak_ by testing modality. Treadmill in the upper panel, and cycle ergometry in the lower panel. Each age-group corresponds to the given decade, not the exact age. Some data collected from graphs or adapted from similar age-groups.

Over the last decades a number of studies have reported reference data on VO_2peak_ by age from cross-sectional studies. Meta-analyses in men have shown annual declines of 0.40 mL/kg/min for both active and sedentary men [12], and 0.44 and 0.35 mL/kg/min for active and sedentary women, respectively, thus assuming linear declines [13]. Similarly, a wide variety of regression equations for predicting VO_2peak_ by age and sex have been published as previously summarized in extensive reviews [9,10], typically reporting declines between 0.3 and 0.5 mL/kg/min per year. In line with this the expected decline in VO_2peak_ is generally accepted to be linear and about 10% per decade [11], although some reports have highlighted geographical differences and nonlinear declines [14]. Notable studies from several countries reporting directly measured VO_2peak_ by gas-analysis during maximal exercise is shown in Fig. 1 and Table 1 [8,[15], [16], [17], [18], [19], [20], [21], [22], [23], [24], [25], [26], [27], [28], [29], [30], [31], [32], [33], [34]], indicating more or less constant (linear) declines. The age-related declines are similar across both cycle ergometry and treadmill exercise, although values from treadmill exercise are generally higher [34]. Although the baseline fitness level decreases with higher age, meaning that percentage decline increases, most studies still have reported declines of 10–15% in older age-groups as well [8,15,16,21,[23], [24], [25],^32^,^33^].

**Table 1:** Key characteristics of cross-sectional studies.

Study	Participants	Characteristics	Country	Modality
Aspenes et al., 2011	2263 Men 2368 Women	Health-survey participants from HUNT aged 20–90 years free from cardiovascular, pumonary and malignant disease.	Norway	Treadmill
Dourado et al., 2021	518 Men 777 Women	Adults >18 years without cardiopulmonary disease, locomotor disorders, ECG abnormalities or other reasons for not performing physical exercise safely.	Brazil	Treadmill
Edvardsen et al., 2013	394 Men 365 Women	Participants aged 20–85 years where participants with either two or more cardiovascular risk factors combined with an age >50 years or with a BP > 180/110 mm Hg were excluded.	Norway	Treadmill
Fleg et al., 2005	435 Men 375 Women	Health-survey participants from the BLSA age 21–87 years without significant cardiopulmonary disease or major orthopedic/neurological disability.	US	Treadmill
Herdy et al., 2011	2388 Men 1564 Women	Exercise tests from a large referral center for cardiology excluding individuals with any symptom of disease or pathology, athletes, smokers, on any medication or BMI≥30. Averaged for active and sedentary individuals.	Brazil	Treadmill
Hollenberg et al., 1998	408 Men 583 Women	Older adults aged 55 years or older without cardiac, cerebrovascular and muskuloskeletal disease able to perform treadmill testing.	US	Treadmill
Inbar et al., 1994	1424 Men	Participants from periodic medical examinations with CPET. Participants with abnormal ECG tracings, or a medical history or physical or laboratory findings of cardiac, respiratory, metabolic or neuromuscular diseases were excluded.	Israel	Treadmill
Kaminsky et al., 2022	9564 Men 6714 Women	Participants aged 20–89 years without known pre-existing diagnosis of cardiovascular disease or chronic obstructive pulmonary disease.	US	Treadmill
Nelson et al., 2010	816 Men	Executive or administrative workers excluding those with hypertension or abnormal pulmonary function.	Canada	Treadmill
Paterson et al., 1999	152 Men 146 Women	Independently living older adults aged 55–85 years regarded medically and physically eligible/able to perform exercise testing.	Canada	Treadmill
Rossi Neto et al., 2019	12,552 Men 5634 Women	Participants coming for check-up or fitness evaluation excluding those with altered ECG or specific medications (beta blockers, medications for chronic obstructive pulmonary disease, or antiarrhythmics).	Brazil	Treadmill
Sanada et al., 2007	656 Women 807 Men	Fitness club members aged 20–80 years without medications or cardiovascular, pulmonary or metabolic disease, or electrolyte abnormalities.	Japan	Treadmill
Grigaliuniene et al., 2013	91 Men 77 Women	Currently healthy, non-smoking participants aged 20–60 years not receiving medical therapy that could affect cardiorespiratory function. No chronic disease or any orthopedic problems prohibiting exercise testing. Baseline resting BP < 140/90 mmHg. Professional athletes were also excluded.	Lithuania	Cycle
Hakola et al., 2011	672 Men 677 Women	Participants from a randomized controlled trial on the health effects of regular physical exercise and diet aged 55–74 years without cardiovascular disease or cancer.	Finland	Cycle
Kaminsky et al., 2017	1717 Men 2777 Women	Participants aged 20–79 years without known pre-existing diagnosis of cardiovascular disease or chronic obstructive pulmonary disease.	US	Cycle
Koch et al., 2009	253 Men 281 Women	Representative sample of adults aged 20–79 years without cardiac disease, pulmonary disease, neuromuscular or musculoskeletal disorders, anemia and use of medications affecting cardiopulmonary function.	Germany	Cycle
Rapp et al., 2018	6462 Men 3628 Women	Mainly participants from workplace health promotion programmes from white-collar occupations aged 21–83 years. Excluding those with contraindications for exercise testing due to acute illness (infections, hypertensive crisis e.g.).	Germany	Cycle
Singh et al., 1989	167 Men	Free from cardiovascular or respiratory disease with a normal physical examination and ECG.	Malaysia	Cycle
Triantafyllidi et al., 2021	118 Men 76 Women	Apparently healthy participants aged 15–69 years after a comprehensive health check-up.	Greece	Cycle
Van der Steeg & Takken 2021	3671 Men 941 Women	Exercise testing data from 11 centres excluding participants with BMI >30, athletes, and smokers.	Netherlands/Belgium	Cycle
Wagner et al., 2021	264 Men 238 Women	Healthy participants aged 20–100 years with a BMI <30 and being a permanent nonsmoker or ex-smoker for at least 10 years. Participants without chronic disease precluding exercise including among others cardiovascular disease, cancer, diabetes and Alzheimers disease.	Switzerland	Cycle

## 2 Age-related declines in longitudinal studies

**Fig. 2:**
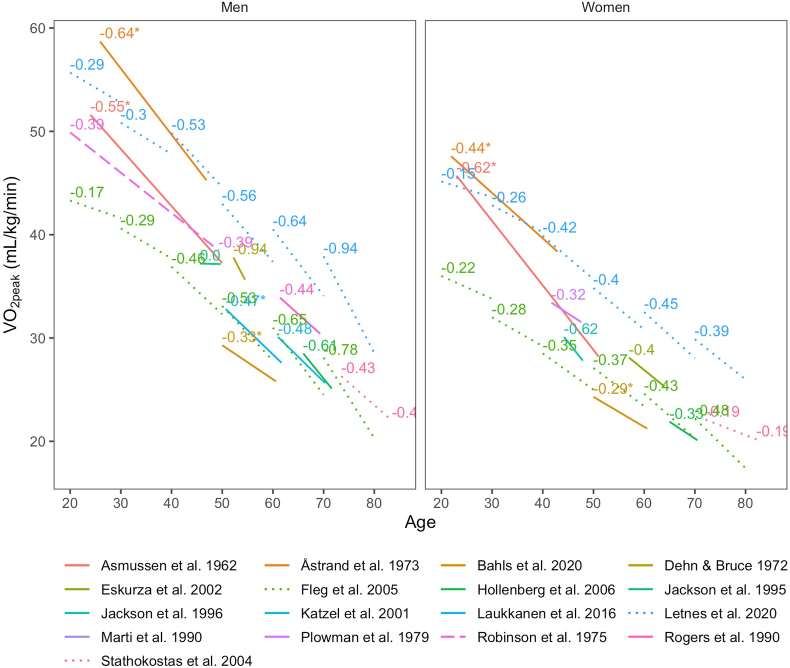
Overview of longitudinal declines in VO_2peak_ from various studies on non-athletes. Mean age at first measurement and length of follow-up for each study is depicted by the start and length of the given lines, respectively. The average annual change is denoted in text with corresponding colour as the given study. Some data are extracted from figures in corresponding publications, and thus may be somewhat inaccurate. The study by Plowman et al., 1979 reported values by different age groups, but values were pooled due to low numbers in several groups. Annotation * = cycle ergometry. (For interpretation of the references to colour in this figure legend, the reader is referred to the Web version of this article.)

**Fig. 3:**
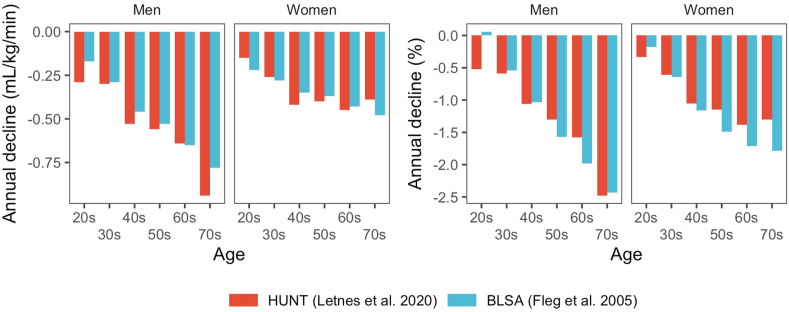
Comparison of data on absolute (left panel) and percentage (right panel) declines in VO_2peak_ from the HUNT (Letnes et al., 2020) and BLSA (Fleg et al., 2005) studies.

Quite a few studies have reported longitudinal data on VO_2peak_, although most have had relatively few participants, narrow selection criteria, a limited age-span, and importantly, data have not been reported stratified by age groups. Generally, it should be noted that cross-sectional studies typically have reported data on several thousands of participants, while longitudinal studies have smaller sample sizes in the tens or hundreds (Table 1, Table 2). Age-related longitudinal declines from several notable studies are summarized in Fig. 2 [16,[35], [36], [37], [38], [39], [40], [41], [42], [43], [44], [45], [46], [47], [48], [49], [50]]*.* The studies by Fleg et al. including 375 women and 435 men from the Baltimore Longitudinal Study of Aging (BLSA) [16] and a study from our group on ∼1500 participants (51% women) from the Trondelag Health Study (HUNT) in Norway [45] both included participants from a wide age range and reported data by ten-year age-groups. Although the estimated longitudinal declines from many of the longitudinal studies vary due to small samples and different inclusion criteria (Table 2, Fig. 2), the findings from the larger BLSA and HUNT studies show the same patterns with increasing declines in both absolute values (mL/kg/min) and percentage VO_2peak_ with higher age, with similar estimates for the declines (Fig. 3). The absolute decline for women in the HUNT Study seemed to level off after 60 years at about 15% decline per decade, but increased towards 20% per decade at high age in the BLSA. In men over 70 years of age the decline approached 25% per decade in both studies. Thus, the decline in both women and men is non-linear throughout life, but based on these two large studies it is clear that this is more pronounced in men than women. The mechanism or explanation for the apparent sex-differences in age-related declines in VO_2peak_ needs further study. Data from the randomized controlled Generation 100 Study following 1567 men and women age >70 years for five years showed annual declines in VO_2peak_ of about 2% after the first year of intervention for both the supervised exercise groups and the control group instructed in national physical activity recommendations. This equated to a 20% ten-year decline despite preserved exercise volumes throughout the study [51].

**Table 2:** Key characteristics of longitudinal studies.

Study	Participants (n)	Baseline age (years)	Follow-up (years)	Sex	Characteristics	Country	Modality
Asmussen et al., 1962	25	24	26	Men	Well-trained physical education students.	Denmark	Cycle
	11	23	28	Women			
Åstrand et al., 1973	35	21.9	21	Women	Physical education students.	Sweden	Cycle
	31	25.9	21	Men			
Bahls et al., 2020	353	50	10.6	Men	Participants in the Study of Health in Pomerania without pulmonary disease.	Germany	Cycle
	335	50	10.6	Women			
Dehn & Bruce 1972	40	52.2	2.3	Men	Healthy men.	US	Treadmill
Eskurza et al., 2002	8	57	7	Women	Healthy, 40–78 years.	US	Treadmill
Fleg et al., 2005	375	48.6	8.3	Women	Participants in the BLSA study without clinically significant cardiovascular or orthopedic/neuromuscular disease.	US	Treadmill
	435	51.9	7.9	Men			
Hollenberg et al., 2006	339	65	5.5	Women	Health-survey participants >55 years without cardiovascular disease or musculoskeletal impairment.	US	Treadmill
	253	66	5.6	Men			
Jackson et al., 1995	156	45.6	4.1	Men	Healthy NASA employees 25 to 70/64 years.	US	Treadmill
Jackson et al., 1996	43	44.2	3.7	Women			
Katzel et al., 2001	47	61	9.3	Men	Healthy volunteers aged 50–79 years.	US	Treadmill
Laukkanen et al., 2016	579	50.7	11	Men	Representative sample of men living in Kuopio participating in the Kuopio Ischaemic Heart Disease Risk Factor study aged 42–60 years.	Finland	Cycle
Letnes et al., 2020	743	48.6	10.2	Women	Adults without cardiovascular, pulmonary or malignant disease at first exercise test participating in the HUNT study.	Norway	Treadmill
	728	50.2	10.2	Men			
Marti et al., 1990	23	19.7	15	Men	Healthy, untrained men who had volunteered for a randomized short-term training study.	Switzerland	Treadmill
Plowman et al., 1979	36	41.7	5.9	Women	Healthy women without hypertension from the general population.	US	Treadmill
Robinson et al., 1975	37	20	29.3	Men	College/University students.	US	Treadmill
Rogers et al., 1990	14	61.4	7.9	Men	Initially healthy aged 37 to 84 years.	US	Treadmill
Stathokostas et al., 2004	34	63.5	10	Men	Random sample aged 55–85 years healthy at both visits.	Canada	Treadmill
	28	62.0	10.1	Women

## 3 Comparison of cross-sectional and longitudinal studies

**Fig. 4:**
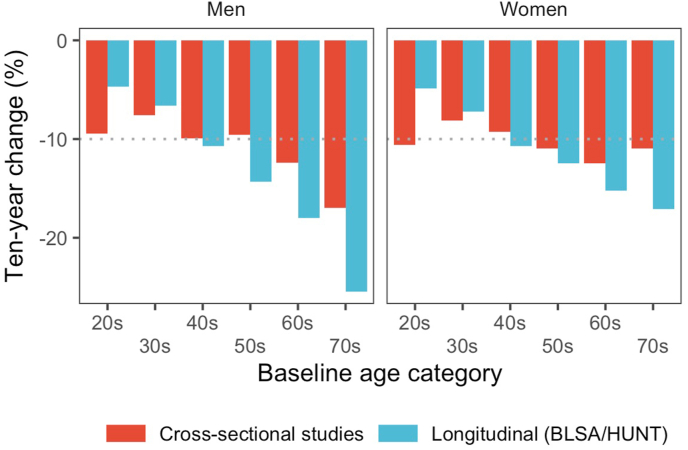
Comparison of cross-sectional and longitudinal age-related declines in VO_2peak_.

The age-related decline estimated from cross-sectional studies compared to findings from the two large longitudinal studies reporting data by age-groups is shown in Fig. 4. When comparing cross-sectional and longitudinal age-related declines the estimates are similar in the middle-aged, but differ at higher age with longitudinal declines being consistently larger (Fig. 4). At younger ages the cross-sectional estimates tend to be higher. In addition to the longitudinal data from BLSA and HUNT presented in Fig. 3 only a few other studies have reported both longitudinal and cross-sectional data on VO_2peak_ in older adults over 60–70 years of age. Stathokostas et al. found 16% and 9% ten-year declines in 34 men and 28 women over 70 years of age, keeping in mind that the sample was relatively small. Also in this study cross-sectional declines were lower than longitudinal declines, although the difference was small in women. In the few studies reporting age-related declines in VO_2peak_ by both cross-sectional and longitudinal measures the mean longitudinal decline is consistently larger than the cross-sectional estimates [8,16,38,[40], [41], [42], [43],^45^,^47^,^50^]. The only exception is the study by Jackson et al. [41] which may be explained by a large self-selection between the first and second measurement as only 10% returned to repeated testing as well as that the cohort itself consisted of highly selected and healthy National Aeronautics and Space Administration (NASA) employees mainly in their middle age. Also, they increased their activity level and decreased body mass during follow-up. Hollenberg et al. examined 592 adult men with a mean age 65 years with repeated measurements of VO_2peak_ over a minimum of three visits and a mean follow-up of 6.3 years [40]. In their repeated measures model they showed that the magnitude of estimates for longitudinal declines were consistently lower than for the cross-sectional estimates, with −0.39 vs −0.23 mL/kg/min/year for women, and −0.69 vs −0.34 mL/kg/min/year for men, in line with the findings from BLSA and HUNT.

## 4 Explanations for the discrepance between cross-sectional and longitudinal studies

The discrepancy between findings using cross-sectional and longitudinal designs is likely to stem from issues regarding selection and survivor bias (i.e. those living until older age most likely were healthier in their younger years than those becoming ill or dying at a younger age). This is especially important given the strong associations between VO_2peak_ and numerous health outcomes, including longevity. This will have a pronounced effect in older ages as the selection pressure due to disease, ailments, and declining PA levels most likely will lead to super-healthy older adults participating in cross-sectional studies. In longitudinal studies this will also theoretically lead to some underestimation of the true age-related decline, as one would expect sicker individuals to experience faster declines in VO_2peak_ and also have a higher risk of mortality or other disease limiting participation in studies requiring maximal exercise to measure CRF. Thus, due to the strong association to health-outcomes for VO_2peak_ and the described survivor bias it is likely that a participant in its eight decade in a cross-sectional study on average would have had higher VO_2peak_ than a 30-year-old in the same study when at the same age, as pointed out also by Fleg et al. [16]. Similarly, cohort effects may play a role in explaining lower declines found in cross-sectional studies. This is evident as studies have shown how CRF has declined on the populational level over the last decades for example in the adult Swedish workforce [52], US youth [53], and children and adolescents from 19 different high-income and upper middle-income countries [54]. Also, the same trend has been shown in US adults from the 1970s to the 2000s, with some evidence of an increase in the 2010s, with these trends linked to changes in body mass index [55]. Pooled data from eight high- and upper-middle-income countries showed a 1.6% decline in CRF per decade from the 1960s until 2016 [56].

Longitudinal, repeated observations are therefore necessary to obtain reliable estimates on declines in VO_2peak_ associated with aging [11,16,38]. Aging itself, but also increasing risk of ceasing or reducing physical activity with higher age is possible explanations behind the accelerating decline in VO_2peak_ with higher age, although this is still not well understood. Based on e.g. longitudinal studies on athletes Hawkins and Wiswell proposed that not only aging itself but inability to maintain exercise training with higher age is also responsible for the accelerated decline seen at older age [11]. Although not elaborated on here it should be noted that statistical model choices and artifacts from various models may also explain differences between crossectional and longitudinal designs [57]. The differences in sample sizes between cross-sectional and longitudinal studies should be kept in mind as well when interpreting differences across these designs. Furthermore, there is also a lack of data regarding differences across ethnicities. These limitiations highlight some of the further research opportunities in the field.

## 5 Clinical implications and concluding remarks

Age is a strong determinant of CRF, and the decline in CRF increases with higher age to 15–20% per decade for women and 20–25% per decade for men after 70 years of age. Age-related declines are consistently larger from longitudinal studies compared to cross-sectional studies possibly due to survivorship bias in the latter. When following patients over time and interpreting trajectories of CRF one should make these assessments in light of findings from longitudinal studies, and not only from findings in cross-sectional studies.

## Author statement

JML designed the study, performed analyses and data visualization, and drafted the first draft. BMN and UW designed the study and revised the draft.

## Funding

The work was funded by The Liaison Committee for Education, Research and Innovation in Central Norway. There are no relations to industry associated with this work.

## Declaration of competing interest

The authors declare that they have no known competing financial interests or personal relationships that could have appeared to influence the work reported in this paper.
